# Characteristics of mycological criteria for the diagnosis of invasive mold infections in patients with severe burn injury

**DOI:** 10.1128/jcm.00950-25

**Published:** 2026-02-05

**Authors:** Emmanuel Dudoignon, Benjamin Deniau, Sorel Tsague, Samia Hamane, Benoit Plaud, Alexandre Mebazaa, Marc Chaouat, Blandine Denis, Francois Dépret, Alexandre Alanio, Sarah Dellière

**Affiliations:** 1 Department of Anesthesiology and Critical Care and Burn Unit, Saint-Louis-Lariboisière Hospital, Université Paris-Cité, Assistance Publique-Hôpitaux de Paris 26930, Paris, France; 2 Lariboisière Hospital, Institut National de la Santé et de la Recherche Médicale (INSERM), UMR-S 942 Mascot 27102, Paris, France; 3 FHU PROMICE, Paris, France; 4 Institut National de la Santé et de la Recherche Médicale (INSERM), PARCC UMR 970 27102, Paris, France; 5 Parasitology-Mycology Department, Hôpital Saint-Louis Paris, Assistance Publique-Hôpitaux de Paris 26930, Paris, France; 6 Plastic Surgery Department, Saint-Louis Hospital, Assistance Publique- Hôpitaux de Paris, Université Paris Cité 26930, Paris, France; 7 Infection Disease Department, Saint Louis Hospital, Assistance Publique-Hôpitaux de Paris 26930, Paris, France; 8 Translational Mycology Research Group, Mycology Department, Institut Pasteur, Université Paris Cité, National Reference Center for Invasive Mycoses and Antifungals 27058, Paris, France; 9 Immunology of Fungal Infection, Institut Pasteur, Université de Paris Cité 27058, Paris, France; University of Calgary, Calgary, Alberta, Canada

**Keywords:** Invasive fungal disease, burn wound, critical care, diagnosis

## Abstract

Invasive fungal diseases (IFDs) are common and often fatal in severe burn patients due to skin barrier loss and immune dysfunction. However, current definitions of invasive mold infections are poorly adapted to this population. This study evaluated the characteristics of various diagnostic criteria and their combinations in relation to clinical outcomes in burn patients. We conducted a retrospective cohort study of all patients admitted to the Burn ICU from 2014 to 2023 with ≥15% total burn surface area and at least one sample sent to the mycology lab. Criteria included direct microscopy, culture (respiratory, skin, or tissue), species-specific quantitative PCR (qPCR) (*Aspergillus*, Mucorales, and *Fusarium*) on plasma/tissue/bronchoalveolar lavage fluid, and serum galactomannan. Among 276 patients, 489/6,184 samples were positive, including 281 skin biopsies (direct examination and conventional culture) and 132 plasma specimens (qPCR). Positive diagnostic criteria ≥1 was found in 93 patients (33.7%): *Aspergillus* (25.7%), Mucorales (10.9%), and *Fusarium* (9.8%). Twenty-seven patients (9.8%) had ≥2 criteria involving ≥2 mold types. Mortality rose with the number of positive criteria: 12.7% (0), 10.7% (1, 2), 27.3% (3, 4), and 46.7% (≥5) (*P* < 0.001). Plasma qPCR was positive in 81.3% of Mucorales, 40% of *Aspergillus*, and 15.4% of *Fusarium* cases with skin involvement. Skin biopsies (direct examination and conventional culture) combined with species-specific plasma qPCR enhance timely and reliable IFD diagnosis in burn patients. Mortality correlated with the number of positive criteria and coexistence of multiple mold species, underscoring the need for broad antifungal coverage and the value of multi-criteria diagnostics to guide treatment.

**IMPORTANCE:**

Invasive mold infections are frequent and often fatal complications in patients with severe burns, occurring in up to 20% of cases with a total burn surface area exceeding 15%. Despite their severity, no standardized case definition currently exists to guide research or clinical management in this population. The performance of existing mycological diagnostic criteria remains unknown in burn patients. In this 10-year retrospective study, we evaluated the diagnostic performance of individual and combined mold-related criteria in relation to patient outcomes, analyzing more than 6,000 clinical samples. These findings provide a first comprehensive assessment of mold diagnostic markers in the burn population.

## INTRODUCTION

Invasive fungal diseases (IFDs) are frequent and often fatal, particularly in patients with severe burns (1). Recent data suggest that patients with >15% total burn surface area (TBSA) have a 26.4% cumulative incidence of proven/putative fungal infection, with 36.8% mortality in probable IFD cases versus 15.3% in those without IFD (2). Burn patients are prone to two main IFDs. Invasive candidiasis, typically candidemia, results from flora translocation due to increased intestinal permeability (e.g., mesenteric ischemia), burn- or catheter-related skin barrier disruption, and/or broad-spectrum antibiotics. Invasive mold infections (IMIs) occur when saprophytic fungi (*Aspergillus* spp., Mucorales, and *Fusarium* spp.) colonize necrotic burn tissue or damaged airways post-smoke inhalation.

International consensus definitions classify IFDs as *proven*, *probable*, or *possible* to harmonize research and clinical practice. *Proven* infection, independent of host factors, requires histological evidence of invasive hyphae or a positive culture from a sterile site. For *probable* and *possible*, the EORTC/MSGERC criteria (3) apply to severely immunocompromised patients, while the Invasive Fungal Diseases in Adult Patients in Intensive Care Unit (FUNDICU) definitions (4) extend to ICU populations with specific host factors such as cirrhosis or viral acute respiratory distress syndrome (ARDS). However, none of these classifications encompass burn patients or their diagnostic specificities (3, 4). Despite local efforts, no globally accepted IMI definition exists in burn patients. Histopathology remains the diagnostic gold standard (i.e., invasive hyphae, small-vessel thrombosis, ischemic necrosis, inflammation in unburned tissue, and/or hemorrhage in uninjured tissue) (5) but is impractical for timely treatment decisions in burn intensive care unit (BICU) (5). Necrotic lesions alone do not confirm diagnosis (6–8). Fungal spores are ubiquitous, complicating interpretation of positive skin cultures. Tools like quantitative PCR (qPCR) and fungal antigen detection in blood may suggest invasiveness, but comprehensive mycological assessment is lacking in burn patients. Given the high mortality, worse outcomes may reflect disease (i.e., invasion) rather than colonization. This study aims to evaluate the performance of different diagnostic criteria and combinations in relation to outcomes in severe burns, following an approach previously applied in ICU settings lacking a gold standard (9, 10).

## MATERIALS AND METHODS

### Patient and study design

**Fig 1:**
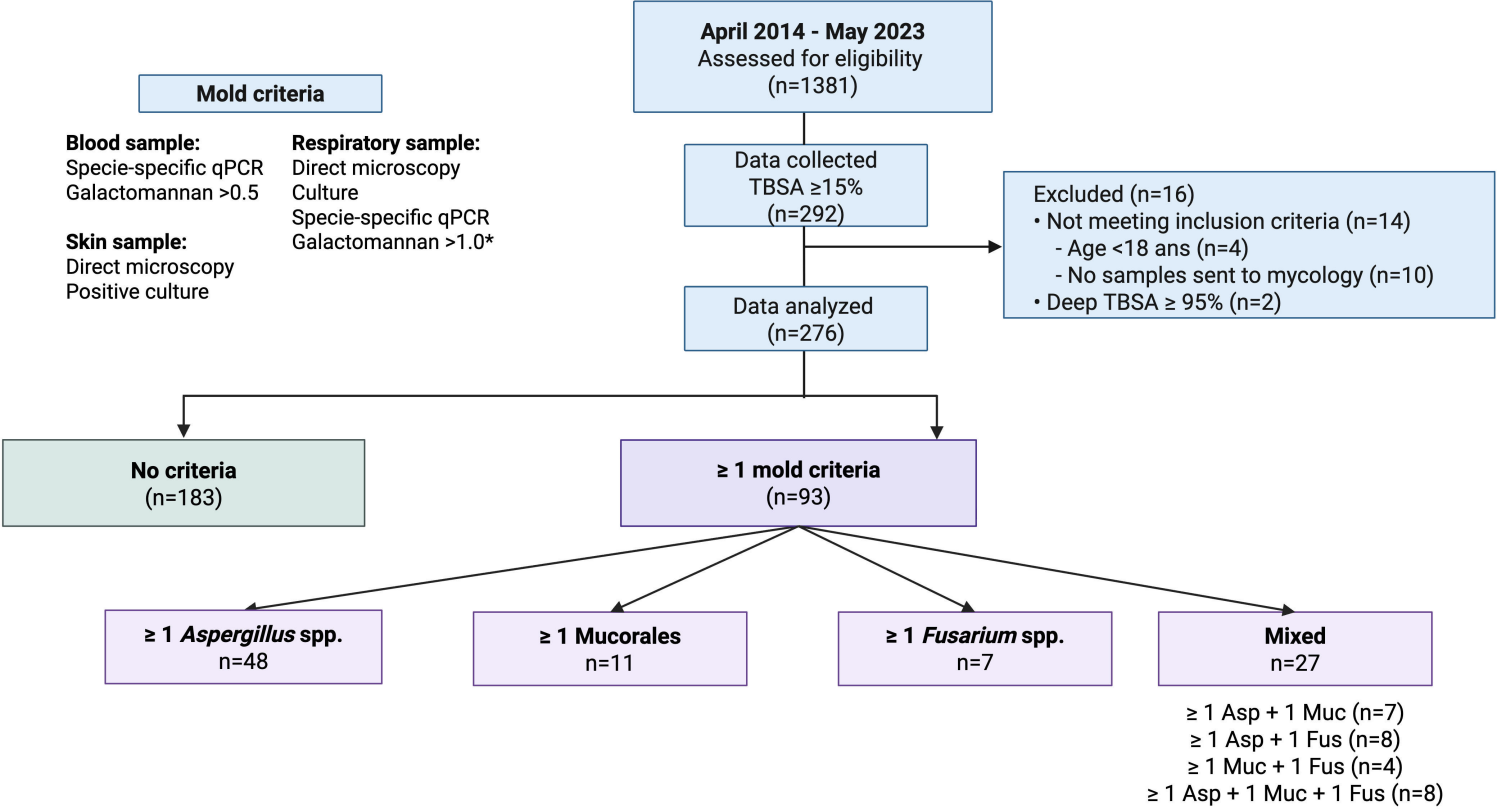
Flowchart of the study. Asp: *Aspergillus* spp.; Muc: Mucorales species; fus: *Fusarium* spp.; TBSA: total burn surface area *only performed in BAL fluid. Created with BioRender.com.

**Fig 2:**
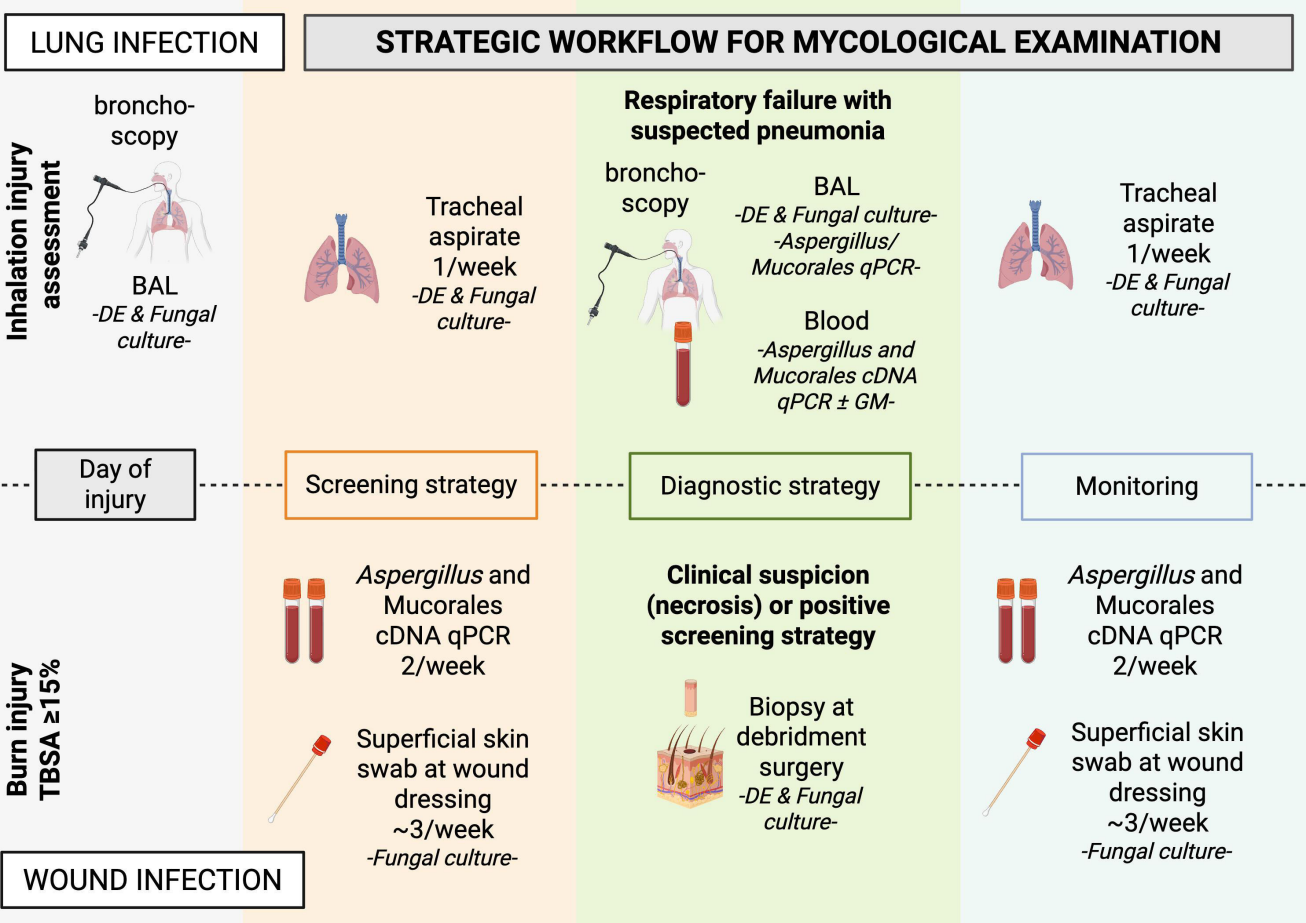
Diagnostic and screening algorithm for burn patients. On the day of the burn, if smoke inhalation is suspected, bronchoscopy and BAL are performed to assess the stage of inhalation injury. The BICU screening strategy includes weekly tracheal aspirates in mechanically ventilated patients and twice-weekly blood samples for circulating Mucorales and *Aspergillus* DNA detection. Superficial skin swabs are collected at each dressing change, typically every 2 days, sent to the bacteriology department and transferred to the mycology department if a fungus grows within 5 days. In the event of respiratory deterioration during ICU stay, bronchoscopy with BAL is repeated to evaluate for pneumonia. If circulating fungal DNA is detected, antifungal therapy is initiated, and any clinically suspicious necrotic skin lesions are further investigated through sampling or biopsy followed by surgical debridement. Ongoing monitoring includes daily dressing changes with inspection for new lesions and continued biweekly blood sampling for circulating fungal DNA. BDG and GM testing were performed at the discretion of the treating physician and were not part of a standardized protocol. Note: The blood qPCR screening program began in January 2015. The *Fusarium*-specific qPCR assay was introduced and made available at our center starting in 2018. This assay was performed only if skin biopsy was positive, to assess invasiveness. cDNA: circulating DNA; DE: direct examination; TBSA: total burn surface area. Created with BioRender.com.

A retrospective cohort study included all patients with severe burns (≥15% TBSA) admitted to the BICU at Saint-Louis tertiary hospital between April 2014 and March 2023. Inclusion required at least one clinical sample sent to the mycology department. Exclusion criteria were age under 18 and burns involving ≥95% TBSA. The study flowchart is shown in Fig. 1. Figure 2 outlines the standardized mycology sampling protocol. Appropriate antifungal management was defined as antifungal therapy combined with surgical debridement, following a previously described strategy (2). This study adheres to STROBE (Strengthening the Reporting of Observational studies in Epidemiology) guidelines (11).

### Mycological criteria testing

Mycological analyses were conducted on blood, bronchoalveolar lavage (BAL) samples, tracheal aspirate, skin biopsy, and skin swab. Sample processing details and tests are provided in Table S1. The following assays were considered mycological criteria if positive: direct microscopy on respiratory sample and skin biopsy, culture of respiratory sample, biopsy or skin swab, pathogen-specific qPCR on respiratory sample or plasma, and galactomannan (GM) on serum. Pathogen-specific qPCR assays (*Aspergillus fumigatus*, Mucorales, and *Fusarium*) were performed on plasma and BAL samples (12–14). Tissue qPCR was not performed routinely due to the high risk of contamination, the inability to distinguish colonization from disease, and the lack of standardized quantification. Positive ß-D-glucan (BDG), when performed, was not included in criteria analysis but studied separately. Positive culture and pathogen-specific qPCR were considered pathogen-specific criteria. Positive GM was considered *Aspergillus*-specific criteria.

### Statistical analysis

Data were reported as percentages, medians, and interquartile ranges (IQRs) [Q1–Q3]. Univariate analyses used Fisher’s exact, chi², and Wilcoxon tests as appropriate. A two-tailed *P* < 0.05 was considered significant. Mortality was analyzed with Kaplan-Meier curves and log-rank tests. Cox models were adjusted for multiple testing and TBSA and age as previously identified as the main factors associated with mortality in this cohort (2). Landmark analysis excluded patients who died before the 25th percentile of time to first positive mycological sample (>7 days). Correlations were performed using Spearman’s correlation test. Analyses were conducted using R software.

## RESULTS

### Population and sample characteristics

The patients’ characteristics are summarized in Table 1. Briefly, 276 patients for whom 6,184 samples were received by the mycology department were included. Patients were mainly males (63.4%) with a median age of 48 years [IQR, 34–61]. A median of 17 [IQR, 7–31] samples were received per patients. Twenty-six (9.4%) patients presented with clinical suspicion of IMI-based mold-suggestive lesions (e.g., necrosis, black eschar, and delayed healing). The time from burn injury to the first positive mycological sample, reflecting the onset of colonization or disease, varied by mold type: 16 [IQR: 8–31], 11 [8–20], and 11 [9–19] days for *Aspergillus* spp., Mucorales, and *Fusarium* spp., respectively. Overall, 93 patients (33.7%) met at least 1 mycological criteria including 71 (25.7%) for A*spergillus* spp., 30 (10.9%) for Mucorales, 27 (9.8%) isolated for *Fusarium* spp. (Fig. S1). A total of 27 patients (9.8%) had ≥2 mycological criteria from ≥2 mold genera (Fig. 1). Number of positive mycological criteria positively correlated with TBSA (*r* = 0.38, 95% confidence interval 0.12–055, *P* < 0.001).

**TABLE 1:** Patient characteristics^*a*^

Characteristics	Patients (*N* = 276)			Samples (*N* = 6,184)
Age (yrs), median [Q1 to Q3]	48 [34 ; 61]			
Male, *n* (%)	175 (63.4%)			
TBSA, median [Q1 to Q3]	33 [25 ; 50]			
ABSI, median [Q1 to Q3]	8 [6 ; 10]			
Smoke inhalation, *n* (%)	103 (37.6)			
Death, *n* (%)	59 (21.4)			
Sample per patient, median [Q1 to Q3]	17 [7 ; 31]			
Type of samples, *n* (%)				
Respiratory*, n* (%)				395 (6.4%)
BAL, *n* (%)				233 (3.8%)
Tracheal aspirate, *n* (%)				162 (2.6%)
Cutaneous*, n* (%)				3,287 (53.2%)
Skin biopsies, *n* (%)				1,777 (28.7%)
Skin swabs, *n* (%)				1,510 (24.4%)
Blood sample*, n* (%)				2,502 (40.4%)
Patients with positive mold criteria				
	*Aspergillus* spp.	*Mucor* spp.	*Fusarium* spp.	
Time from burn injury to the first positive mycological criteria (days)	qPCR: 25 [10 ; 37] Skin biopsy culture: 18 [8 ; 29] BAL or TA culture: 13 [7 ; 34] GM serum: 30 [29 ; 32] GM BAL: 11 [8 ; 16]	qPCR: 11 [8 ; 22] Skin biopsy culture: 11 [9 ; 16]	qPCR: 18 [17 ; 22] Skin biopsy culture: 11 [9 ; 18]	
1 criterion, *n* (%)	40 (14.5%)	14 (5.1%)	12 (4.3%)	
2 criteria, *n* (%)	13 (4.7%)	3 (1.1%)	8 (2.9%)	
≥3 criteria, *n* (%)	18 (6.5%)	13 (4.7%)	7 (2.5%)	
Number of patients with only positive cutaneous cultures, *n* (%)	30 (10.8%)			
Number of patients with positive blood qPCR, *n* (%)	56 (20.3%)			
Number of patients with ≥1 mold type criteria (mixed)	27 (9.8%)			
Number ofpatientst with ≥1 respiratory criterion (respiratory sample with positive culture, qPCR and/or GM)	16 (5.8%)			

^*a*^ ABSI, abbreviated burn severity index; GM, galactomannan; PCR, polymerase chain reaction, Q1, Quartile 1; Q3, Quartile 3; TBSA, total burn surface area.

A total of 65 patients (23.6%) had at least 1 cutaneous sample positive for at least 1 mold species (Fig. S1). Patients clinically suspected of wound IMI had a median of 3 (range, 1–9) positive mycological criteria compared to 0 (range, 0–7) in patients without clinical suspicion (*P* < 0.0001). Thirty (10.9%) patients had only a positive skin biopsy culture for *Aspergillus* spp., Mucorales, and *Fusarium* spp. The positivity rate for different sample types is detailed in Table 2. Positive criteria breakdown according to the type of mold and type of sample is shown in Table 3. Skin biopsies were associated with more positive tests than swabs (281/1,777 (15.8%) vs 42/1,510 (2.8%), *P* < 0.001). A total of 219 (79.3%) patients had at least 1 plasma qPCR performed, 56 (20.3%) of whom had at least 1 positive qPCR. GM and BDG testing were performed only 22 and 12 times, respectively, as they are not part of standardized management protocol. The positivity rates were 13.6% and 83.3%, respectively. Serum GM was tested in three patients, two of whom had ≥1 positive results, both associated with a concomitant positive plasma *Aspergillus*-qPCR. Additional details on positive GM are provided in Table S2.

**TABLE 2:** Positivity rate to assess test yield

	Test performed (*n*)	Positive tests (*n*)	Positivity rate (%)
Respiratory samples			
BAL	233	13	5.6%
GM	94	5	5.3%
Tracheal aspirate	162	3	1.9%
Cutaneous samples			
Skin biopsies	1,777	281	15.8%
Skin swabs	1,510	42	2.8%
Blood samples			
qPCR	2,468	132	5.3%
GM	22	3	13.6%
BDG	12	10	83.3%

**TABLE 3:** Breakdown of mold-specific positive mycological results by sample type and mold type (*Aspergillus*, Mucorales, and *Fusarium*)^*a*^

	*Aspergillus* spp.	Mucorales	*Fusarium* spp.
Blood sample	68 plasma qPCR 3 GM	57 plasma qPCR	7 plasma qPCR
Skin sample	150 skin samples: 116 biopsy cultures 34 swab cultures	85 skin samples: 82 biopsy cultures 3 swab cultures	124 skin samples: 115 biopsy cultures 9 swab cultures
Respiratory sample	16 respiratory samples^*b*^: 3 qPCR 10 cultures 5 GM	0 respiratory samples: 0 qPCR 0 cultures	1 respiratory sample: 0 qPCR 1 cultures
Total positive sample	237 positive samples	142 positive samples	132 positive samples

^*a*^ qPCR, quantitative polymerase chain reaction; GM, galactomannan.

^*b*^ The sum of individual tests (18) exceeds the total number of samples (16) because certain samples underwent multiple testing modalities (e.g., culture and GM/qPCR performed on the same specimen).

Sixteen (5.8%) patients had at least 1 respiratory sample positive with a mycological criterion (i.e., direct microscopy, culture, GM, or qPCR). Their clinical, radiological, and mycological features are detailed in Table 4. Of these, 12 (75.0%) had only a single positive criterion per respiratory sample, and just 1 patient (6.3%) had a concomitant positive serum GM or concordant blood qPCR suggesting colonization in most cases.

**TABLE 4:** Characteristics of patients and samples with a positive respiratory sample^*a*^

Patient ID number	Positive sample	TBSA (%)	Inhalation injury	Stage inhalation	ARDS (within 7 days of burn and/or inhalation)	Tomodensitometry (as close as possible to the respiratory sample)	Time to positivity of respiratory sampling after burn injury (days)	DM	Culture positive	qPCR (ct) respiratory	GM serum (index)/plasma qPCR	GM respiratory (index)	Positive GM respiratory/total sampled	Outcome	Number of positive lung/blood criteria
1’	BAL	37	No	NI	Yes	No pulmonary infiltrates Condensation No cavitation	16	N	Negative	NA	NA/negative	Positive 6.53	1 / 1	Alive	1
2’	BAL	90	Yes	3	Yes	Pulmonary infiltrates Condensation No cavitation	22	N	Negative	NA	NA/negative	Positive 1.06	1 / 2	Dead	1
3’	BAL	32	Yes	ND	Yes	Pulmonary infiltrates Condensation No cavitation	10	N	Negative	Negative	NA/negative	Positiv**e** 2.55	1 / 5	Alive	1
4’	BAL	40	No	NI	Yes	NA	11	N	Negative	Negative	<0.5/negative	Positive 1.56	1 / 1	Dead	1
5’	BAL	18	Yes	2	No	Pulmonary infiltrates Condensation No cavitation	2	N	Negative	Negative	NA/negative	Positive 2.47	1 / 4	Alive	1
6	BAL	60	Yes	2	Yes	Pulmonary infiltrates Condensation No cavitation	4	N	*Aspergillus versicolor*	NA	NA/positive *A. fumigatus*	< 1.0	0 / 1	Dead	2
7	BAL	66	Yes	2	Yes	Pulmonary infiltrates Condensation Cavitation	6	N	*A. fumigatus* *Alternaria* sp.	NA	NA/positive *A. fumigatus*	< 1.0	0 / 1	Alive	2
8	BAL	50	No	NI	Yes	Pulmonary infiltrates Condensation No cavitation	6	N	*Aspergillus niger*	NA	NA/positive *A. fumigatus*	NA	0 / 0	Alive	2
9	BAL	39	Yes	1	Yes	Pulmonary infiltrates Condensation No cavitation	91	N	*Aspergillus versicolor*	NA	<0.5/positive *A. fumigatus*	NA	0 / 0	Dead	2
10	BAL	18	No	NI	Yes	Pulmonary infiltrates Condensation No cavitation	5	P	*A. fumigatus*	Positive 36.9	<0.5/negative	<1.0	0 / 1	Dead	3
11	BAL	35	No	NI	Yes	Pulmonary infiltrates Condensation No cavitation	38	N	*Aspergillus ustus*	Negative	NA/positive *A. fumigatus*	<1.0	0 / 4	Alive	2
12	BAL	30	No	NI	Yes	NA	17	P	*A. fumigatus*	Positive 35.5	NA/negative	<1.0	0 / 6	Alive	3
13	BAL	15	No	NI	Yes	Pulmonary infiltrates Condensation Cavitation	7	N	*A. fumigatus*	Positive 32.9	NA/negative	<1.0	0 / 1	Alive	2
14	TA	60	No	NI	Yes	Pulmonary infiltrates Condensation No cavitation	12	P	*A. fumigatus*	NA	NA/NA	NI	NI	Dead	2
15	TA	46	Yes	2	Yes	Pulmonary infiltrates Condensation No cavitation	37	N	*Aspergillus flavus*	NA	NA/NA	NI	NI	Alive	1
16	TA	45	Yes	2	Yes	Pulmonary infiltrates Condensation No cavitation	42	N	*Fusarium* sp.	NA	NA/positive *A. fumigatus*	NI	NI	Alive	2

^*a*^ TBSA, total burn surface area; ARDS, acute respiratory distress syndrome; DM, direct microscopy; GM, galactomannan; BAL, bronchoalveolar lavage; TA, tracheal aspirate; ND, not determined; NA, not assesed; NI, no inhalation injury.

### Association of multiple positive criteria from different samples

Among patients with clinical suspicion of IMI (*n* = 26), culture of skin biopsy was positive in 38.5% of cases for *Aspergillus* sp. (*n* = 10) and Mucorales (*n* = 10). It was 42.3% (*n* = 11) for *Fusarium* sp. Nineteen patients among those with clinical suspicion of IMI had at least 1 positive PCR, of which 11 (42.3%) were positive for *Aspergillus*, 12 (46.2%) were positive for Mucorales, and 3 (11.5%) were positive for *Fusarium*.

Among patients with skin samples (swabs or biopsies) positive for *Aspergillus* sp. (*n* = 45), 18 (40%) had at least 1 positive plasma *Aspergillus*-specific qPCR. For Mucorales, 13/16 (81.3%) patients with a positive skin sample had at least 1 positive plasma Mucorales-specific qPCR. In contrast, only 4/26 patients (15.4%) with a positive culture for *Fusarium* spp. had a positive plasma *Fusarium*-specific qPCR.

One hundred and forty-eight (53.6%) direct microscopy exams were positive among the 281 culture-positive skin biopsies. At least one skin culture and/or plasma *Aspergillus*-specific PCR was systematically positive (100%) in the case of positive serum GM (*n* = 2) (Table S2).

### Evaluation of mycological criteria in relation to patient mortality

**Fig 3:**
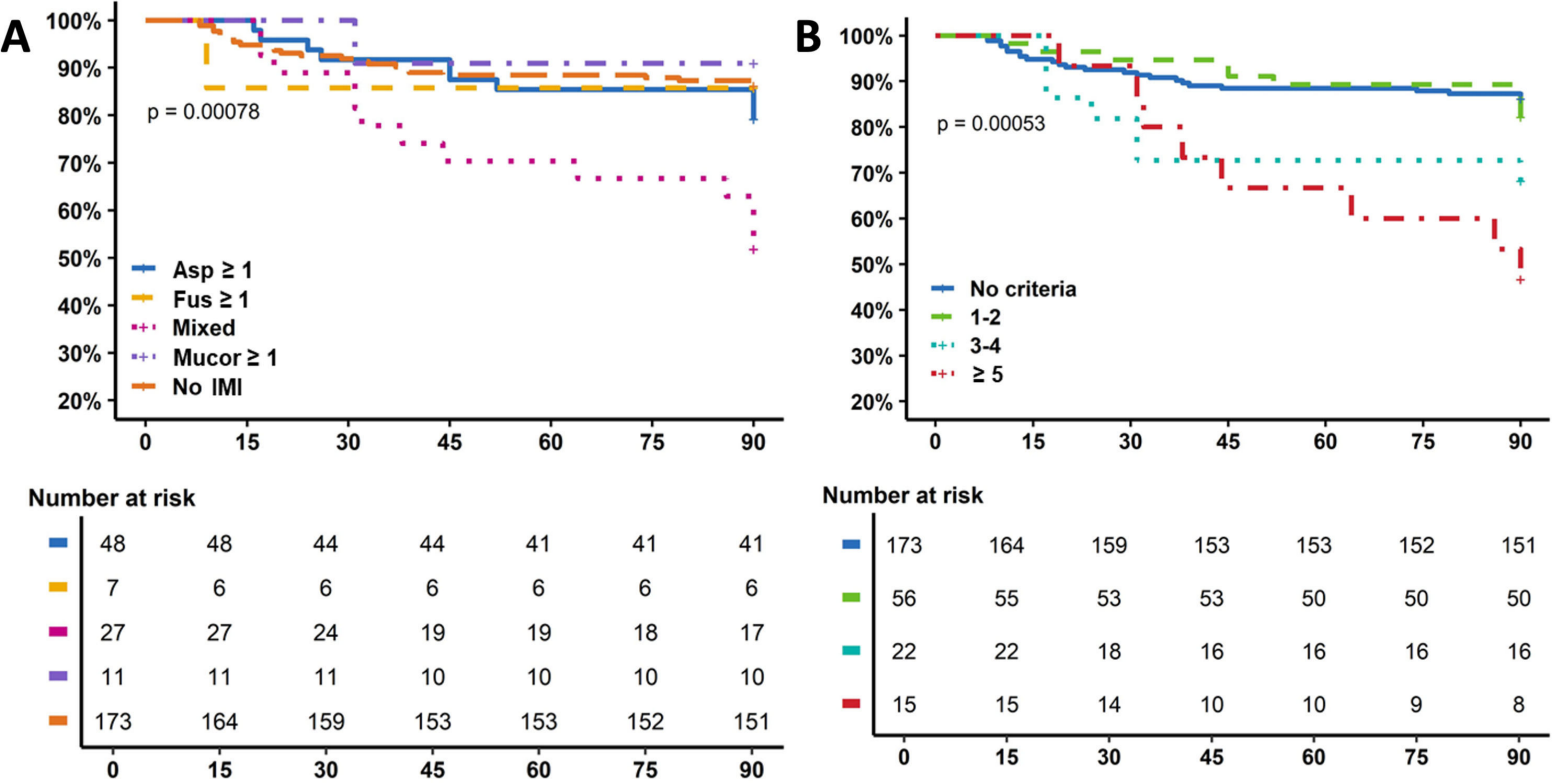
Adjusted survival curves according to (**A**) the type of IMI and (**B**) total IMI criteria – landmark analysis D7. Asp, *Aspergillus*; Fus, *Fusarium;* Mucor, Mucorales.

The number of samples taken did not differ between living and deceased patients (17 [7–30] vs 16 [4–32], *P* = 0.55). Raw mortality curves according to the type of mold criteria and number of criteria are shown in Fig. S2. Considering that the median time from burn injury to first positive criteria was 12 [IQR, 8–25] days, a landmark analysis was performed at day 7 excluding patients who died early due to extreme severity of the burn. As shown by the survival curves (Fig. 3A) according to the positivity of at least 1 criterion (*Aspergillus*, Mucorales, *Fusarium,* and mixed), there was a significant increased mortality (37%) observed for patients with mixed criteria. For all IMI mycological criteria, mortality was significantly different in patients with 0, 1–2, 3–4, and ≥5 mycological criteria with death rates of 22 (12.7%), 6 (10.7%), 6 (27.3%), and 7 (46.7%), respectively (*P* < 0.001) (Fig. 3B; Fig. S4).

Mortality was significantly higher when criteria were ≥3 for *Aspergillus* sp. (odds ratio [OR] 3.2 [1.2–8.6], *P* = 0.01), Mucorales (OR 3.4 [1.1–10.5], *P* = 0.02) and *fusarium* (OR 4.0 [1.4–12.0], *P* = 0.007). The cumulative number of criteria ≥5 was independently associated with mortality with adjusted OR 2.67 [1.12–6.35], *P* = 0.027 (Table S3 and S4).

**Fig 4:**
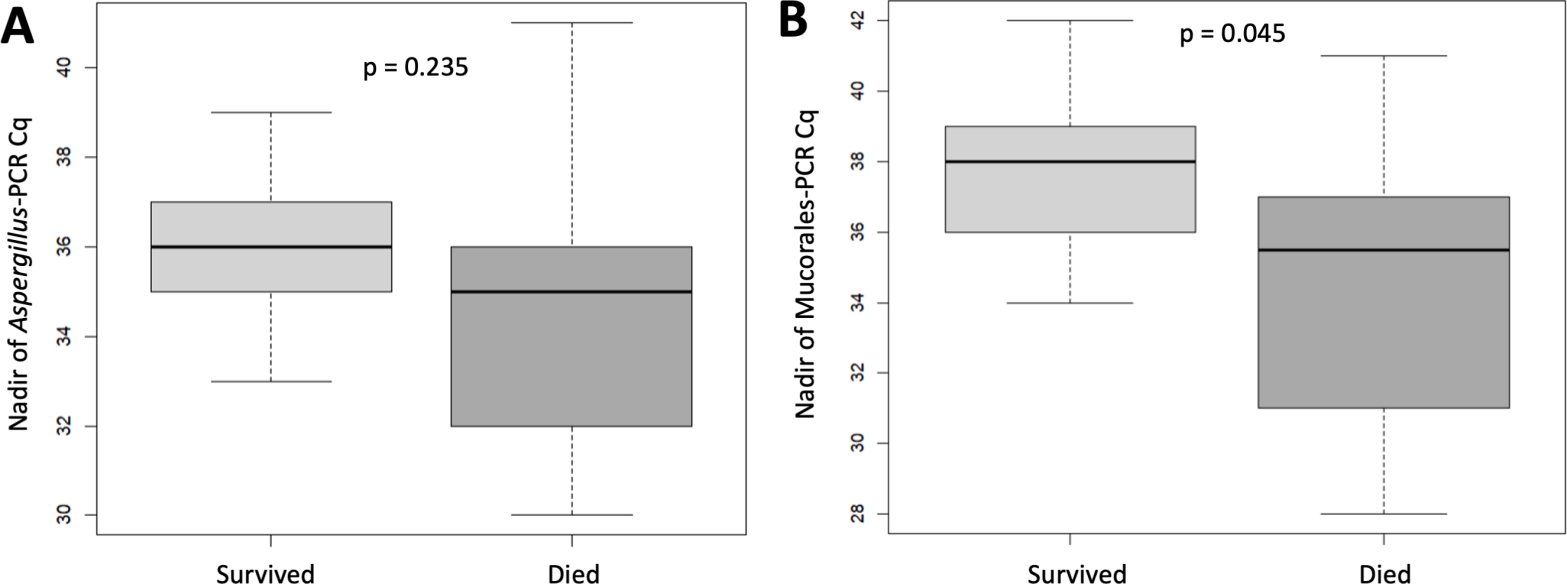
Nadir plasma fungal load of *Aspergillus* (**A**) and Mucorales (**B**) by patient outcome.

For all species, the D-90 mortality of patients with a culture-positive skin biopsy did not differ according to the plasmatic qPCR result (negative 7/30 [23.3%] versus positive 9/34 [26.5%]). There was a trend toward lower mortality when the skin biopsy was negative with a positive plasmatic qPCR (1/22 [4.5%], *P* = 0.26) (Fig. S3). Patient mortality was not statistically different in the case of negative culture and positive qPCR versus positive culture and negative qPCR for *Aspergillus* sp. (4/20 [20%] vs 8/26 [30.8%], *P* = 0.410) and Mucorales (4/14 [28.6%] vs 2/3 [66.7%], *P* = 0.210). Plasma Cq values were significantly lower in deceased patients (36 [32–37] vs 38 [36–39], *P* = 0.045) for Mucorales, and no difference was observed for *A. fumigatus* (35 [32–36] vs 36 [35–37], *P* = 0.235) as shown in Fig. 4. Among patients with at least one positive skin biopsy, a positive direct microscopy was significantly associated with higher mortality (48.3%) compared to a negative result (19.4%; *P* < 0.005). Notably, time to treatment did not differ significantly between deceased and surviving patients (4 [3–7] vs 3 [1–5] days, *P* = 0.16). Receiving operating characteristic (ROC) curve predicting mortality based on the number of blood qPCRs, direct microscopy, skin biopsy culture, and the combination of skin biopsy culture with plasma qPCR is shown in Fig. S5.

### Characteristics of mycological criteria as prognosis marker

Time from first positive to first negative PCR was numerically longer in deceased patients than in survivors for both *A. fumigatus* (9 [4–11] vs 4 [3–8] days, *P* = 0.23) and Mucorales (8 [4–20] vs 5 [4–10] days, *P* = 0.24).

## DISCUSSION

In this large, homogeneous 10-year retrospective cohort of 276 severely burned patients (TBSA ≥ 15%), we evaluated the diagnostic and prognostic value of multiple mycological criteria, including conventional methods (e.g., culture and direct microscopy), molecular tools (specific qPCR) and antigen testing across 6,184 skin, respiratory, and blood samples. Findings suggest that skin biopsies, rather than swabs, combined with blood qPCR provide a reliable, faster alternative to histopathology. Although no difference in mortality was observed between mold species when at least one criterion was positive, the total number of criteria was independently associated with mortality, highlighting the potential utility of combining diagnostic criteria to guide treatment decisions.

### Disease vs colonization

A major challenge in severe burn patients at risk for IMI is distinguishing disease (i.e., invasion) from colonization. Mycological criteria were met in 33.7% of patients; due to their environmental ubiquity and possible deposition during injury or care, positive cultures are frequent. We previously identified risk factors, such as confined space burns, suggesting exposure to aerosolized spores during the fire (2). While histopathology remains the gold standard, faster, reliable diagnostics are needed. Clinical suspicion alone, despite routine full burn exams, often misses IMI: our prior algorithm identified 31 (11.2%) IMI cases without clinical suspicion, with a median of 3 (range, 2–7) positive mycological criteria (2). Overall, positive predictive value increases with the number of positive criteria and defining a threshold, accepting some false positives given the poor prognosis of IMI, could help guide timely antifungal treatment.

Few studies have included Mucorales-specific plasmatic qPCR in their diagnostic algorithm (1, 2, 15), though its performance evaluation remains limited in this population. Legrand et al. (16) demonstrated that detection of circulating Mucorales DNA enables earlier diagnosis (by 4.5 to 15 days) and treatment, correlating with reduced mortality. We found that when a skin biopsy culture is positive, the associated Mucorales-, *Aspergillus*-, and *Fusarium*-specific blood qPCR is positive in 81.3%, 38.5% and 15.4% of cases despite similar detection thresholds of these tools (13, 14, 17). This supports differing invasive potential among molds, with Mucorales exhibiting higher angioinvasion and causing more rapid and extensive necrosis as previously suggested (18). Combining species-specific blood qPCR with biopsy may better reflect invasiveness. Finally, while species-specific qPCR on skin biopsies may over-detect colonization due to its high sensitivity, it could still help quantify fungal burden when cultures are positive.

Our findings indicate that positive mycological criteria in respiratory samples were uncommon and predominantly isolated, even with comprehensive testing. The absence of concurrent positive serum GM or blood qPCR in nearly all cases supports the interpretation that these findings reflect colonization rather than invasive disease. Overall, this suggests that true pulmonary mold infections are likely rare in this patient population.

### Outcome associated with mycological criteria

The number and type of criteria (i.e., positive direct microscopy and lower Mucorales-qPCR Cq value) and mixed mold criteria were associated with higher mortality, reflecting disease invasiveness and fungal burden. These findings have direct clinical implications, as empirical and targeted therapy should ensure sufficient spectrum of activity to cover potential multi-species infections. Such criteria should carry greater weight in future diagnostic algorithms. While the association between an increasing number of positive criteria and mortality likely reflects disease, the diagnostic value of such combinations also depends on the interplay of both positive and negative results. Negative findings may attenuate the overall likelihood of disease, and future diagnostic frameworks should integrate the weighting of both positive and negative mycological criteria to refine predictive accuracy. Similar challenges have been faced in defining diagnostic criteria for COVID-19-associated pulmonary aspergillosis (CAPA), where early proposals suggested combining criteria (19) and assessing them against mortality outcomes (9, 10). In our study, mortality did not differ by fungal species, likely due to some patients meeting only a single criterion, suggesting colonization rather than disease. Direct microscopy positivity in skin biopsy, although never identified as an isolated criterion, was significantly associated with poor outcome, warranting prompt antifungal treatment, even before species-level identification, followed by therapy reassessment.

Interestingly, a surprising trend toward lower mortality in patients with positive plasma qPCR alone (vs. culture positivity) may be attributed to our screening and treatment strategy, in which an early positive plasma qPCR prompts targeted antifungal therapy, a practice previously associated with reduced mortality (2, 16, 20). Plasma qPCR, more indicative of invasiveness, enabled earlier diagnosis when fungal burden was lower (only one or two positive criteria) and earlier treatment, even without biopsy-confirmed infection or clinical suspicion, leading to improved outcomes. The slight difference in Mucorales Cq values between survivors and non-survivors, although confirmed in a bicentric cohort (20), should be interpreted cautiously, as it may reflect minor fungal load variations rather than a biologically meaningful threshold.

### Toward a case definition for IMI in patients with severe burns

Currently, no standardized case definition exists for diagnosing IMI in patients with patient with severe burn injuries. Our findings provide a foundation to identify key diagnostic criteria. As case definitions are primarily developed for clinical research purposes to enhance comparability and reproducibility across studies, they should prioritize specificity over sensitivity. The detection of at least one mycological criterion during screening should prompt thorough clinical assessment and repeated sampling to confirm diagnosis. Criteria should be weighted, with the highest priority given to plasma species-specific qPCR and skin biopsy when used together to increase specificity. Given the lower diagnostic yield and higher risk of representing colonization (positivity rate: 2.8% vs 15.8% for biopsy), skin swabs should not be recommended for IMI diagnosis. GM may be included, although additional data are needed, as its low sensitivity in non-neutropenic populations could limit its contribution; this was also observed in our study without a benefit compared to *Aspergillus*-specific qPCR (21). BDG should be excluded from diagnostic algorithms. Its high positivity rate (83.3%) in our cohort did not correlate with fungal infection or positive mycological criteria and often resulted from inappropriate prescription. Prior studies also report high false-positive BDG rates in burn patients (22, 23).

### Limits of this study

Diagnosing IMI in burn patients remains challenging due to the absence of EORTC/MSGERC host factors, complicating identification of those who might benefit from antifungal therapy. As with studies on CAPA, our analysis is limited by the lack of a definitive gold standard, such as histopathological confirmation of invasive hyphae (10). Assessing criteria in relation to mortality carries bias, as death in this population is often multifactorial, linked to bacterial/fungal infections or burn-related organ failure. This retrospective, single-center study also relied on clinician-driven antifungal treatment and diagnostics. While a center-wide algorithm existed, its application may have varied over time.

### Conclusion

To date, no study has evaluated the diagnostic and prognostic value of multiple mycological criteria across varied sampling methods in such a large burn cohort. Our findings demonstrate that clinical suspicion alone is insufficient for diagnosing IMI; suspicious lesions require targeted sampling. A prospective multicenter study is needed to compare diagnostic criteria, particularly skin biopsy plus plasma qPCR, against histopathology. Establishing a standardized consensus definition for IMI in burn patients is essential to advance research, improve outcomes, and limit overuse of antifungals in this high-risk population.

## SUPPLEMENTAL MATERIAL

Supplemental text jcm.00950-25-s0001.docx STROBE checklist.

Supplemental material jcm.00950-25-s0002.docx Tables S1 to S4; Fig. S1 to S5.

ASM does not own the copyrights to Supplemental Material that may be linked to, or accessed through, an article. The authors have granted ASM a non-exclusive, world-wide license to publish the Supplemental Material files. Please contact the corresponding author directly for reuse.
