# Supplementary material for: Characteristics of mycological criteria for the diagnosis of invasive mold infections in patients with severe burn injury
Source: J Clin Microbiol. 2026 Feb 5;64(3):e00950-25. doi: 10.1128/jcm.00950-25 (PMC12977568; doi:10.1128/jcm.00950-25)
Supplement: Supplemental material — Tables S1 to S4; Fig. S1 to S5. [file jcm.00950-25-s0002.docx]

**Table S1.** Description of Sample Collection, Handling, and Testing Procedures

| **Mycological criteria** | | Plasma | BAL and tracheal aspirate | Skin biopsy | Skin swab | Serum |
| --- | --- | --- | --- | --- | --- | --- |
| Direct microscopy | | - | Performed with Calcofluor White and read at 475nm with a Nikon Eclipse E600 fluorescence microscope | | - | - |
| Culture | | - | Samples were seeded on BBL^TM^ CHROMagar Candida Medium, 2% MALT extract + chloramphenicol agar (VWR Chemical, Leuven, Belgium), Sabouraud supplemented with chloramphenicol/gentamicin (Biorad, Marnes-la-coquette, France) and incubated at 30 and/or 37°C for 3 weeks. | | | - |
| Molecular biology | DNA extraction  WNA together with internal control were extracted using the QIAsymphony DSP virus/pathogen kit (Qiagen, Hilden, Germany) on a QIAsymphony apparatus (Qiagen) | 1mL | bead-beaten pellet re-suspended in 1000µL of DNA-free | Bead-beaten skin biopsy suspended in 1000µL of DNA-free | - | - |
|  | Aspergillus fumigatus qPCR | *Aspergillus fumigatus* specific in-house qPCR [1] | | | - | - |
|  | Mucorales qPCR | Mucorales (*Rhizopus* sp., *Rhizomucor* sp., *Mucor* sp., *Lichtheimia* sp.)  specific in-house qPCR [2] | | | - | - |
|  | Fusarium qPCR | Pan-*Fusarium* specific in-house qPCR [3]  Plasma *Fusarium* qPCR was performed if a skin sample was positive from only 2017. | | | - | - |
| Galactomannan | | - | GM detection was performed using a Platelia Bio-Rad kit (Bio-Rad Laboratories, Hercules, CA) according to the manufacturer. Each positive result was retested the following day with the same sample, as recommended [4]. Only samples found positive twice were considered positive. **ODI of 1** was in BAL. | - | - | GM detection was performed using a Platelia Bio-Rad kit (Bio-Rad Laboratories, Hercules, CA) according to the manufacturer. Each positive result was retested the following day with the same sample, as recommended [4]. Only samples found positive twice were considered positive. **ODI of 0.5** was considered positive in serum. |
| ß-D-glucan | | - | - | - | - | BDG testing was performed using Fungitell assay (Cape Cod Diagnostics) according to the manufacturer’s instructions. BDG was considered positive when >80 pg/mL. |
| Other information | |  | BAL was centrifuged 10 min at 3,000 g. Pellet was separated in 3 portions for direct examination, culture and DNA extraction. |  |  |  |

[1] Alanio A, Menotti J, Gits-Muselli M, et al. Circulating Aspergillus fumigatus DNA Is Quantitatively Correlated to Galactomannan in Serum. Frontiers in Microbiology **2017**; 8:405–8. [2] Millon L, Caillot D, Berceanu A, et al. Evaluation of serum Mucorales PCR for the diagnosis of Mucormycoses: The MODIMUCOR prospective trial. Clin Infect Dis **2022** [3] Dellière S, Guitard J, Sabou M, et al. Detection of circulating DNA for the diagnosis of invasive fusariosis: retrospective analysis of 15 proven cases. Med Mycol **2022**; 60. [4] Guigue N, Lardeux S, Alanio A et al. Importance of operational factors in the reproducibility of Aspergillus enzyme immunoassay. PlOS One **2015** .

BAL: bronchoalveolar lavage; ODI: Optical density index; WNA: whole nucleic acid

**Table S2.** **Focus on positive galactomannan assay (n=8) in 7 patients**

| **Patient ID number** | **Serum GM testing**  **positivity** | **Respiratory GM testing positivity** | **Plasma *Aspergillus* qPCR positivity** | **Respiratory or skin sample** | **Total positive Aspergillus criteria** |
| --- | --- | --- | --- | --- | --- |
| 1’ | NA | 100% (1/1 tested) | 0% (0/6 tested) | Negative BAL  Negative skin biopsies (0/2)  Negative skin swab (0/6) | 1 |
| 2’ | NA | 50% (1/2 tested) | 0% (0/6 tested) | Negative BAL  Negative skin biopsies (0/24)  Negative skin swab (0/26) | 1 |
| 3’ | NA | 20% (1/5 tested) | 0% (0/5 tested) | Negative BAL  Negative skin biopsies (0/1)  Negative skin swab (0/5) | 1 |
| 4’ | 0% (0/2 tested) | 100% (1/1 tested) | 0% (0/5 tested) | Negative BAL  Negative skin biopsies (0/1)  Negative skin swab (0/1) | 1 |
| 5’ | NA | 25% (1/4 tested) | 0% (0/13 tested) | Negative BAL  Positive skin biopsies (2/4)  Positive skin swabs (2/8) | 3 |
| 6’ | 100% (1/1 tested) | NA | 54.5% (6/11 tested) | Negative BAL  Positive skin biopsies 5/25  Negative skin swab 0/3 | 6 |
| 7’ | 100% (2/2 tested) | NA | 6.1% (2/33 tested) | Negative BAL  Positive skin biopsies 5/13  Skin swabs 3/17 | 6 |

**BAL**: Bronchoalveolar lavage; **DE**: Direct examination; **GM**: galactomannan; **NA**: not assesed

**Table S3**. Adjusted odds ratios for D-90 mortality according mold species

|  | **Mortality** | | |
| --- | --- | --- | --- |
| *Predictors* | *Estimates* | *CI* | *p* |
| ≥ 1 *Aspergillus* spp. | 1.18 | 0.55-2.52 | 0.663 |
| ≥ 1 Mucorales | 0.79 | 0.10-5.90 | 0.814 |
| ≥ 1 *Fusarium* spp. | 1.09 | 0.15-8.13 | 0.931 |
| Mixed | 2.43 | 1.15-5.11 | 0.020 |
| Observations 263  R² Nagelkerke 0.201 | | | |

**Table S4**. Adjusted odds ratios for D-90 mortality according number of criteria

|  | **Mortality** | | |
| --- | --- | --- | --- |
| *Predictors* | *Estimates* | *CI* | *p* |
| [1-2] criteria | 1.00 | 0.47-2.13 | 0.994 |
| [3-4] criteria | 2.27 | 0.95-5.43 | 0.065 |
| ≥ 5 criteria | 2.67 | 1.12-6.35 | 0.027 |
| Observations 263  R² Nagelkerke 0.206 | | | |

**Figure S1.** Venn diagram of patients with patients with ≥1 positive mycological criteria according to type of mold criteria or organ involvement

**
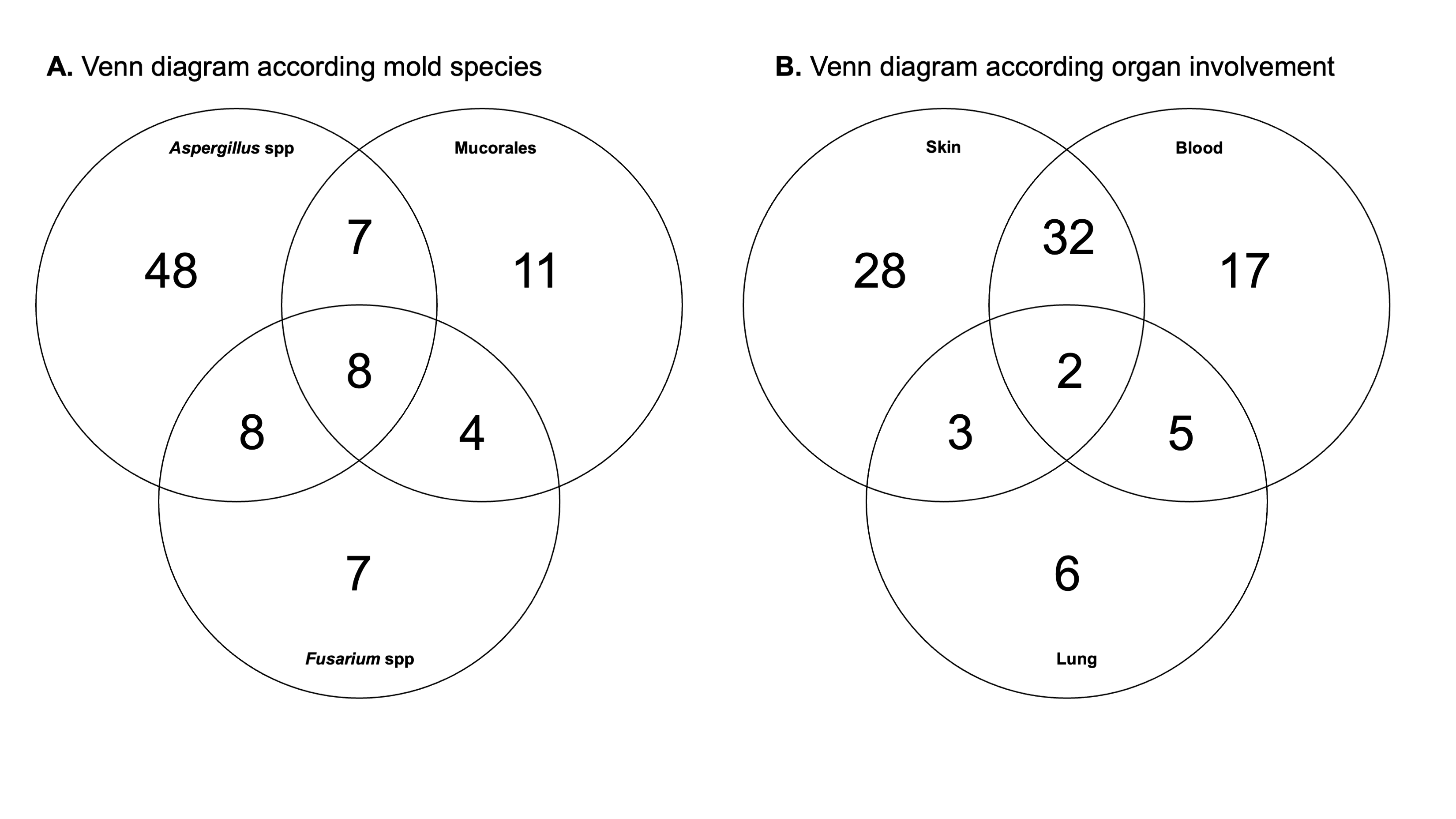
**

**Figure S2.** Raw Survival curves according to the type of mold criteria and number of criteria

**
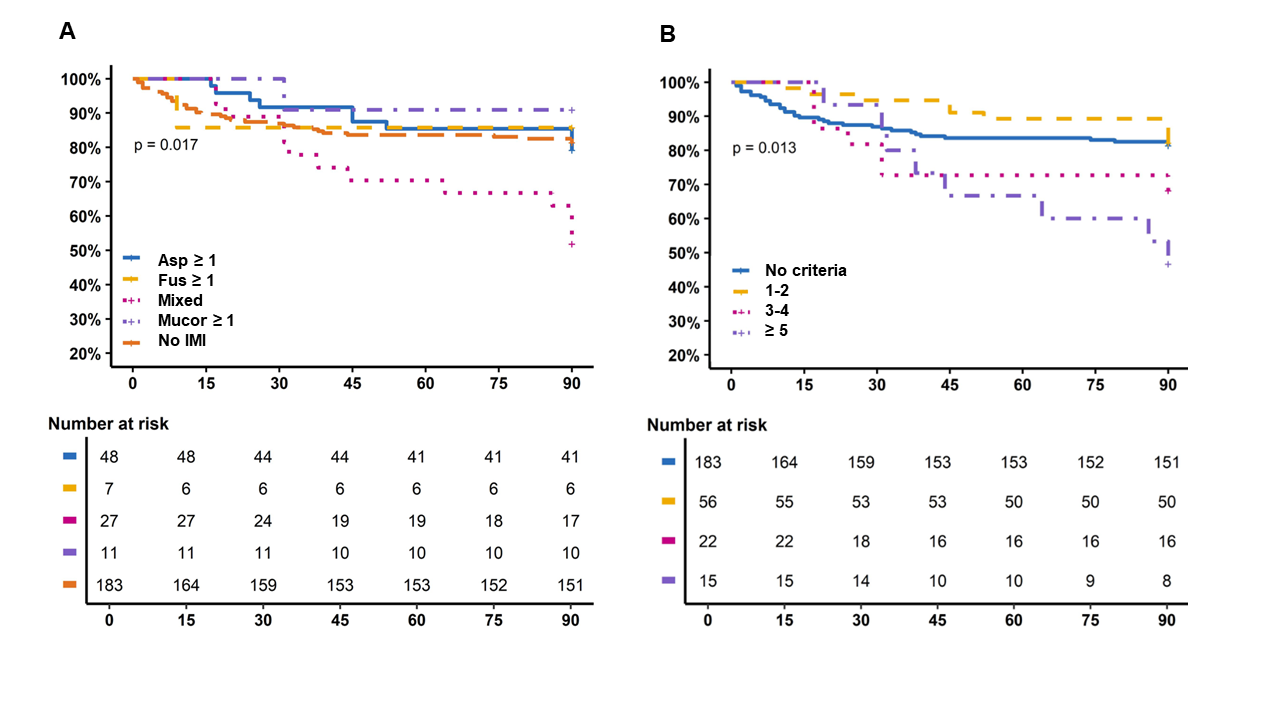
**

**Figure S3.** Survival curves between positivity of biopsies skin culture and plasma qPCR


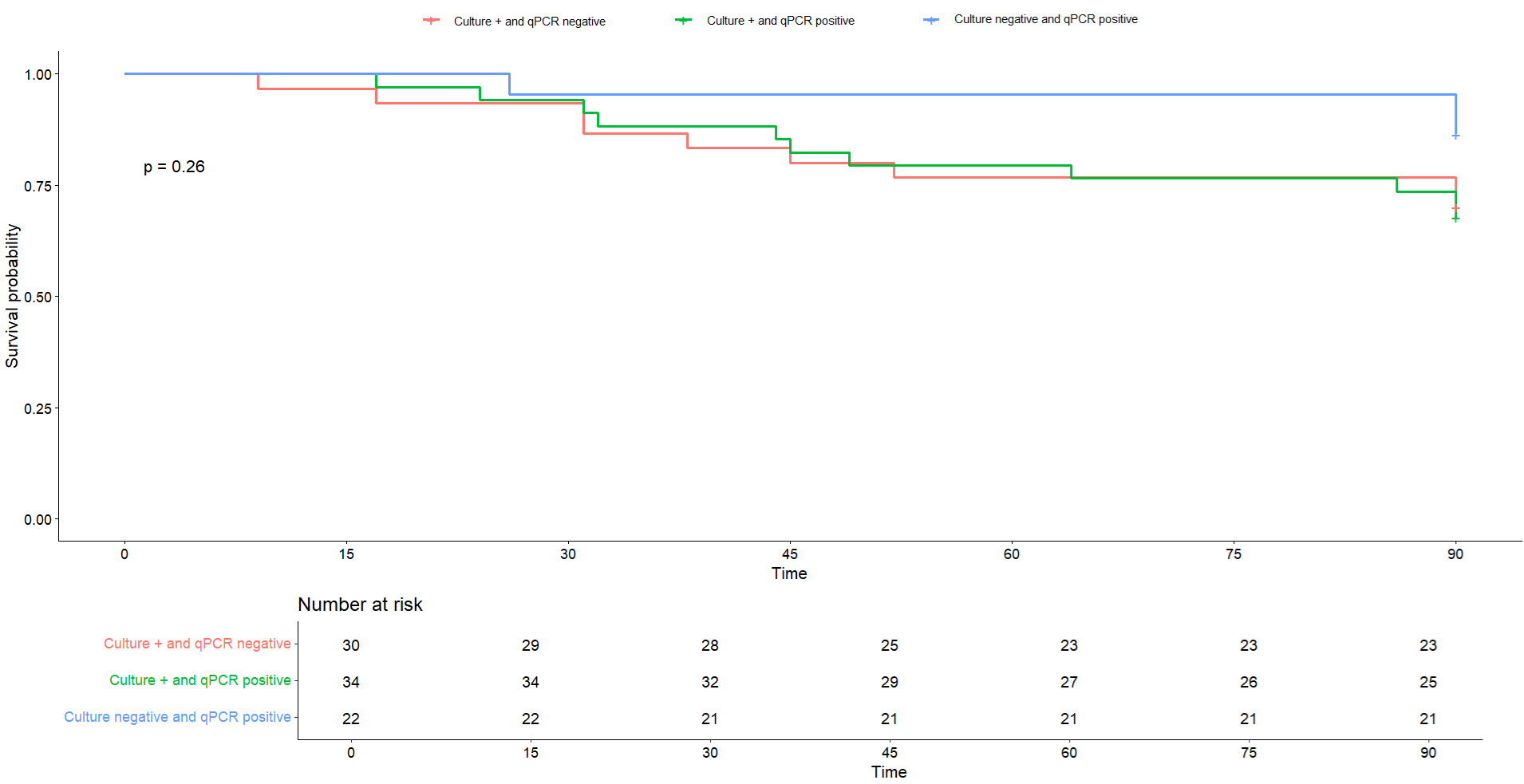


**Figure S4.** Adjusted survival curves according to the total of IMI criteria [0-2]; [3-4] and ≥ 5 – Landmark analysis D7

**
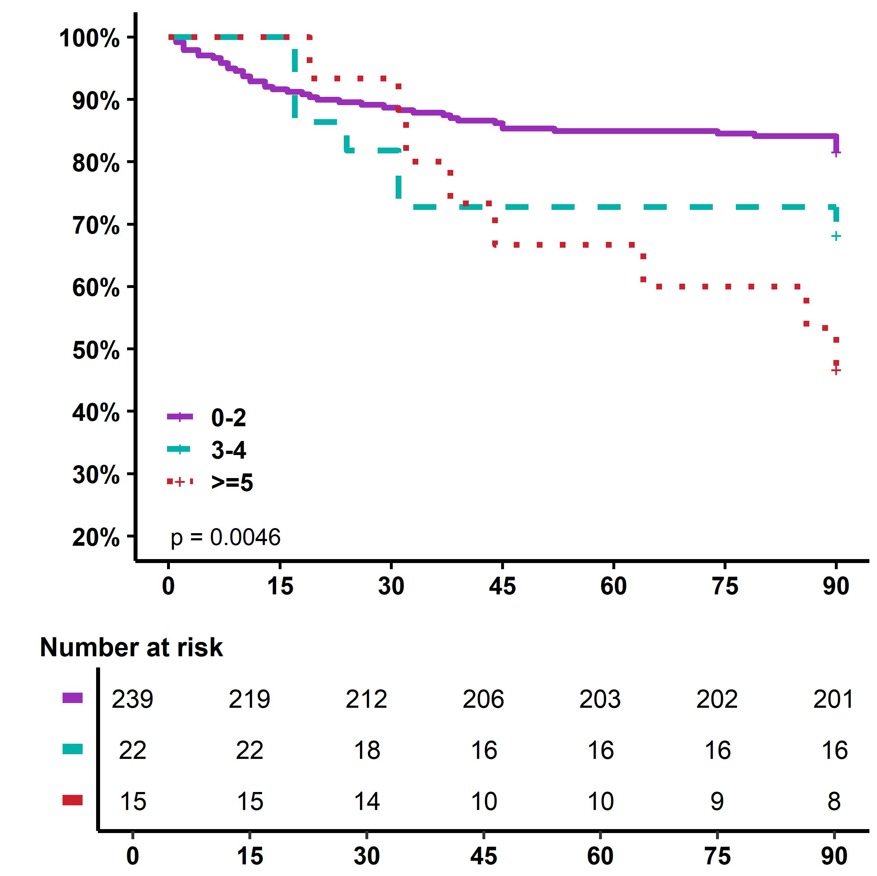
**

**Figure S5.** ROC curves predicting mortality based on the number of blood qPCRs, direct examinations (DE), skin biopsies, and the combination of skin biopsies with plasma qPCR.

**
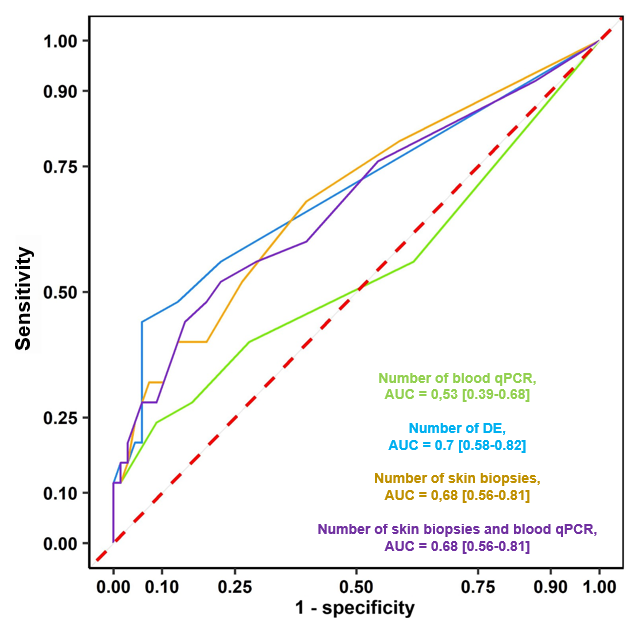
**
